# Supplementary material for: Optical mapping of ground reaction force dynamics in freely behaving Drosophila melanogaster larvae
Source: eLife. 2024 Jul 23;12:RP87746. doi: 10.7554/eLife.87746 (PMC11265794; doi:10.7554/eLife.87746)
Supplement: Supplementary file 1. [file elife-87746-supp1.docx]

**Supplementary File 1: Measured effective Young’s Modulus per elastomer mixture post plasma treatment.**

| **Elastomer Mixture** | **Mean Young’s Modulus (Pa)** | **Plasma power (W)** |
| --- | --- | --- |
| 10% Sylgard®184,  90% Sylgard®527 | 27697.978 ± 913.45 | 30 |
| 6.6% Sylgard®184,  94.4% Sylgard®527 | 25999.17 ± 991.93 | 30 |
| 5% Sylgard®184,  95% Sylgard®527 | 18596.08 ± 186.05 | 30 |
| 100% Sylgard®527 | 12325.35 ± 395.45 | 30 |
| 100% Nusil®GEL8100 | 3340.30 ± 53.45 | 30 |
| 10% Sylgard®527,  90% Nusil®GEL8100 | 8974.32 ± 621.83 | 180 |
| 50% Sylgard®527,  50% Nusil®GEL8100 | 18404.14 ± 439.85 | 180 |
| 90% Sylgard®527,  10% Nusil®GEL8100 | 38387.87 ± 2768.63 | 180 |
